# Docosahexaenoic Acid Attenuates Visceral Pain by Suppressing Spinal CXCL10/CXCR3/ERK Signaling

**DOI:** 10.3390/nu18071113

**Published:** 2026-03-30

**Authors:** Xi Yin, Anqi Jiang, Yu Han, Jianhua Qu, Jianya Zhao, Hao Gong, Xiaorong Luo, Xu Li, Ying Lu

**Affiliations:** Research Institute of Public Health, Nantong University, 9 Seyuan Road, Nantong 226019, China

**Keywords:** visceral pain, docosahexaenoic acid, chemokine, spinal cord, neuron

## Abstract

**Background:** Visceral pain is the primary symptom of functional gastrointestinal disorders, yet its spinal molecular mechanisms remain poorly defined. **Methods:** Using a 2,4,6-trinitrobenzenesulfonic acid (TNBS)-induced chronic inflammatory visceral pain model, the role of the spinal CXCL10/CXCR3/ERK signaling axis and the analgesic effect of docosahexaenoic acid (DHA) were investigated. **Results:** TNBS significantly upregulated CXCL10 and CXCR3 in spinal dorsal horn neurons and increased ERK phosphorylation. Intrathecal CXCL10-neutralizing antibody or CXCR3 antagonist NBI-74330 reduced visceral hypersensitivity and suppressed spinal ERK activation in TNBS mice. Exogenous CXCL10 induced CXCR3-dependent hyperalgesia and ERK phosphorylation in the spinal cord. Intrathecal DHA attenuated TNBS-induced visceral pain, downregulated spinal CXCL10/CXCR3 expression, and inhibited ERK signaling. In Neuro-2a cells, DHA also blocked LPS-induced activation of the same pathway. **Conclusions:** This study suggests that the analgesic effect of DHA may involve the inhibition of the spinal CXCL10/CXCR3/ERK signaling pathway.

## 1. Introduction

Chronic visceral pain, a defining feature of functional gastrointestinal disorders and inflammatory bowel diseases, affects millions worldwide [1]. It is diffuse, poorly localized, and often accompanied by emotional and autonomic disturbances that significantly reduce quality of life [2,3]. Current analgesics offer limited benefit: opioids carry risks of tolerance and dependence, while NSAIDs may worsen gastrointestinal injury [4,5]. Uncovering the underlying mechanisms and identifying new therapeutic targets remain key priorities in visceral pain research.

Central sensitization is a key mechanism driving chronic visceral pain. Spinal inflammatory mediators modulate neuronal excitability and promote hypersensitivity. Chemokines, initially defined by their role in leukocyte trafficking, are now known to mediate neuron–glia signaling in the CNS [6]. Emerging evidence suggests that specific chemokine pathways contribute to pain in a signal- and disease-specific manner [7,8]. Among these, CXCL10/CXCR3 signaling, traditionally associated with immune recruitment, is upregulated in spinal neurons and glia after nerve injury or inflammation [9]. Although implicated in neuropathic pain [10], its role in visceral pain remains poorly characterized. Given the distinct neurobiology of visceral nociception, elucidating CXCL10/CXCR3 involvement is critical to understanding its pathogenesis.

Extracellular signal-regulated kinase (ERK), a Mitogen-Activated Protein Kinase (MAPK) family member, plays a central role in spinal pain plasticity. Injury rapidly induces ERK phosphorylation in the dorsal horn, modulating ion channels, synaptic proteins, and gene expression to promote central sensitization [11]. ERK integrates diverse upstream inputs, including neurotrophic factors, inflammatory mediators, and chemokines. CXCR3, a Gi-coupled receptor, has been shown to activate ERK via Gαi/o and β-arrestin-dependent mechanisms [12]. These findings suggest that the CXCL10/CXCR3/ERK axis could represent a potential signaling cascade in visceral pain regulation.

Omega-3 polyunsaturated fatty acids have drawn interest for their anti-inflammatory and analgesic properties [13]. Docosahexaenoic acid (DHA), the principal omega-3 in neural membranes, exerts neuroprotection via suppression of arachidonic acid metabolism, induction of pro-resolving mediators, and activation of GPR120 [14]. DHA enhances neuronal survival by promoting the synthesis of membrane phosphatidylserine and activating key kinase signaling pathways [15]. Its metabolites, e.g., resolvins, exert anti-inflammatory and antioxidant effects, reducing tissue damage, inflammation, and apoptosis in ischemic and neurodegenerative disease models [16]. Additionally, DHA and its metabolites promote hippocampal neurogenesis and maintain synaptic proteins, thereby exerting protective effects in various neurological diseases [17]. Epidemiological studies report that DHA intake correlates inversely with chronic pain risk and improves symptoms in rheumatoid arthritis and dysmenorrhea [18]. Animal studies further support its role in relieving inflammatory and neuropathic pain [19]. However, its effect on visceral pain and the underlying mechanisms remains poorly defined.

Based on these, it is hypothesized that the spinal CXCL10/CXCR3/ERK pathway contributes to central sensitization in visceral pain, and that DHA exerts analgesic effects by inhibiting this cascade. Using a TNBS-induced experimental colitis model, we examined spinal levels of CXCL10, CXCR3, and phosphorylated ERK, and employed pharmacological approaches to define their roles in visceral nociception. We further evaluated the regulatory effect of DHA on this pathway. This study aims to elucidate the spinal regulatory mechanisms of DHA on visceral pain, providing a molecular target basis for developing nutritional intervention strategies based on omega-3 fatty acids.

## 2. Experimental Section

### 2.1. Animals

Male C57BL/6 mice (18~22 g) were purchased from the Experimental Animal Center of Nantong University (Jiangsu, China). A total of 112 mice were used in this study. They were maintained at a room temperature of 22 ± 1 °C and a 12 h light/dark cycle with free access to water and food. All animals were acclimatized to the housing conditions for at least 7 days before any experimental procedures. All animal procedures were performed according to the guidelines of the International Association for the Study of Pain and approved by the Animal Care and Use Committee of Nantong University.

### 2.2. Induction of Experimental Colitis and Experimental Groups

Experimental colitis was induced as described previously [20]. Briefly, mice were anesthetized with diethyl ether, and a 1.2 mm polyethylene catheter was inserted 4 cm into the colon via the anus. A total of 1.75 mg of TNBS (Sigma-Aldrich, St. Louis, MO, USA) dissolved in 50% ethanol (100 μL) was administered intrarectally. After instillation, mice were kept in a supine position with the lower body elevated for 5 min to prevent reflux. Control animals received an equal volume of vehicle (Normal saline, NS).

To investigate the role of spinal CXCL10/CXCR3 signaling and the modulatory effect of DHA, experiments were performed in five independent batches, each consisting of six mice per group (*n* = 6). The sample size was determined based on effect sizes estimated from pilot studies to ensure the statistical power. The experimental design was as follows: Batch 1 (Model Validation): (1) NS; (2) TNBS model. Batch 2 (CXCL10 Neutralization): (1) TNBS + vehicle (control serum, i.t.); (2) TNBS + CXCL10 neutralizing antibody (1 μg, i.t.); (3) TNBS + CXCL10 neutralizing antibody (10 μg, i.t.). Batch 3 (CXCR3 Antagonism): (1) TNBS + vehicle (2% DMSO in saline, i.t.); (2) TNBS + NBI-74330 (2 μg, i.t.); (3) TNBS + NBI-74330 (20 μg, i.t.). Batch 4 (Exogenous CXCL10 Effect): (1) Sham + PBS (i.t.); (2) Sham + recombinant murine CXCL10 (10 ng, i.t.); (3) Sham + recombinant murine CXCL10 (100 ng, i.t.). Batch 5 (DHA Intervention): (1) TNBS + vehicle (sterile saline, i.t.); (2) TNBS + DHA (1 μg, i.t.); (3) TNBS + DHA (10 μg, i.t.). For intervention groups (Batches 2, 3, and 5), behavioral tests were conducted before the TNBS model induction and on the seventh day after induction. On the seventh day, drugs were intrathecally administered, and behavioral tests were performed at predetermined time points after the administration. For Batch 4, behavioral tests were carried out at predetermined time points (1, 3, and 6 h) following a single intrathecal injection.

Four mice were excluded from the final analysis for reasons unrelated to experimental treatments: one died from accidental suffocation during anesthetic recovery, and three were euthanized due to severe fight-induced injuries sustained during single housing. No unexpected serious adverse events directly attributable to the experimental procedures, including TNBS induction or intrathecal drug administration, were observed.

### 2.3. Visceral Hypersensitivity Testing

Colorectal distension was performed before and 7 days after TNBS instillation as described previously [21]. This time point represents the subacute phase of inflammatory visceral pain (characterized by persistent inflammation and an established phase of stable hyperalgesia) and serves as a suitable window for evaluating the efficacy of drug interventions. A latex double-lumen catheter with a 2 mm balloon was inserted 4 cm into the descending colon and lubricated with vaseline. Distension was achieved by inflating the balloon with 0.1~1.0 mL of air. Mice were placed in small lucite chambers and allowed to awaken and acclimate for at least 30 min before testing. Each distension was repeated three times with more than 5 min intervals for recovery. Abdominal withdrawal reflex was recorded by blinded observers. The visceral pain threshold was defined as the minimum volume of air that elicited a visible abdominal withdrawal reflex, characterized by a rapid and sustained abdominal muscle contraction lifting the abdomen off the platform.

### 2.4. Drugs and Administration

Recombinant murine CXCL10 was obtained from PeproTech (Rocky Hills, NJ, USA). The CXCR3 antagonist NBI-74330 was purchased from Tocris (Warsaw, Poland), and the CXCL10 neutralizing antibody was from R&D Systems (Minneapolis, MN, USA). DHA (sodium salt; Sigma-Aldrich) was dissolved in sterile saline. For intrathecal injection, a spinal puncture was performed between the L5 and L6 vertebrae with a 30-gauge needle to deliver reagents into the cerebrospinal fluid [19,22].

### 2.5. Real-Time PCR

Animals were euthanized under isoflurane anesthesia, and the L6-S1 spinal cord segments were rapidly dissected. Total RNA was extracted using TRIzol reagent (Invitrogen, Carlsbad, CA, USA), and 1 μg of RNA was reverse-transcribed into cDNA using the PrimeScript RT reagent kit (Takara Bio, Shiga, Japan). Quantitative real-time PCR was performed on a Rotor-Gene 6000 system (Corbett Research, Mortlake, NSW, Australia) using SYBR Premix Ex Taq II (Takara Bio). The primer sequences were as follows: Cxcl10 forward, 5′-TGA ATC CGG AAT CTA AGA CCA TCA A-3′ and reverse, 5′-AGG ACT AGC CAT CCA CTG GGT AAA G-3′; Cxcr3 forward, 5′-TAC CTT GAG GTT AGT GAA CGT CA-3′ and reverse, 5′-CGC TCT CGT TTT CCC CAT AAT C-3′; Gapdh forward, 5′-AAA TGG TGA AGG TCG GTG TGA AC-3′ and reverse, 5′-CAA CAA TCT CCA CTT TGC CAC TG-3′. PCR cycling conditions were 95 °C for 30 s, followed by 40 cycles at 95 °C for 5 s, 56 °C for 30 s, and 72 °C for 30 s. Melt curve analysis was conducted to confirm product specificity. Relative gene expression was calculated using the 2-ΔΔCt method with Gapdh as the internal control.

### 2.6. Western Blot

At 7 days after TNBS instillation, animals were transcardially perfused with PBS. The L6-S1 spinal cord segments were collected and homogenized in lysis buffer containing protease and phosphatase inhibitors. Protein concentrations were determined using a BCA Protein Assay Kit (Thermo Fisher Scientific, Hanover, IL, USA). Equal amounts of protein (30 μg per lane) were separated by 10% SDS-polyacrylamide gel electrophoresis and transferred onto PVDF membranes. Membranes were blocked and incubated overnight at 4 °C with primary antibodies against CXCR3 (1:200, rabbit, Boster (Pleasanton, CA, USA)), pERK (1:1000, rabbit, Cell Signaling (Danvers, MA, USA)), and ERK (1:1000, rabbit, Cell Signaling). GAPDH (1:20,000, mouse, Sigma-Aldrich) was used as the loading control. After incubation with horseradish peroxidase-conjugated secondary antibodies, immunoreactive bands were visualized using enhanced chemiluminescence (Thermo Fisher Scientific) and captured on film (EMD Millipore, Burlington, MA, USA). Band intensities were quantified with ImageJ 1.52 software.

### 2.7. Immunohistochemistry

The L6-S1 spinal cord segments were sectioned at 30 μm thickness using a cryostat and processed for immunofluorescence as previously described [23]. The following primary antibodies were used: CXCR3 (rabbit, 1:200, Boster, Wuhan, China), CXCL10 (goat, 1:100, R&D Systems), ionized calcium-binding adapter molecule 1 (IBA-1; goat, 1:1000, Abcam, Waltham, MA, USA; rabbit, 1:3000, Wako, Osaka, Japan), glial fibrillary acidic protein (GFAP; mouse, 1:5000, EMD Millipore), and neuronal nuclear antigen (NeuN; mouse, 1:800, EMD Millipore). Sections were incubated with Cy3- or Alexa Fluor 488-conjugated secondary antibodies (1:1000, Jackson Inc., West Grove, PA, USA). Fluorescent images were acquired using a Leica SP8 confocal microscope (Wetzlar, Germany).

### 2.8. Cell Culture and Treatment

Neuro-2a cells were cultured in Dulbecco’s modified Eagle’s medium (DMEM; Hyclone, Logan, UT, USA) supplemented with 10% fetal bovine serum (FBS; Hyclone), 100 U/mL penicillin, and 100 μg/mL streptomycin (Invitrogen). Cells were stimulated with lipopolysaccharide (LPS) for 6 h. DHA (10 or 30 μmol/L) was added 30 min before LPS stimulation. After incubation, total RNA and protein were extracted for quantitative real-time PCR and Western blot analyses.

### 2.9. Quantification and Statistics

Band densities for pERK (42/44 kDa), ERK (42/44 kDa), CXCR3 (55 kDa), and GAPDH (38 kDa) were quantified using ImageJ software. The same rectangular area was applied to each band, and background values were subtracted. Animals were randomly assigned to experimental groups using a random number table. Behavioral tests were conducted by experimenters blinded to the group allocations. Sample coding for molecular biological analysis was performed by an independent individual, and all data collection and analysis were completed by the analysts before unblinding. Data are presented as mean ± SEM. Behavioral data were analyzed by two-way repeated-measures ANOVA followed by Bonferroni post hoc tests. qPCR data were analyzed by one-way ANOVA with Bonferroni correction, and differences between the two groups were assessed using Student’s *t*-test. Statistical analyses were performed using the SPSS PRO online tool (https://www.spsspro.com/, access date 20 March 2026), and *p* < 0.05 was considered statistically significant.

## 3. Results

### 3.1. TNBS Induces Neuronal CXCL10 Upregulation Predominantly in Spinal Dorsal Horn

**Figure 1:**
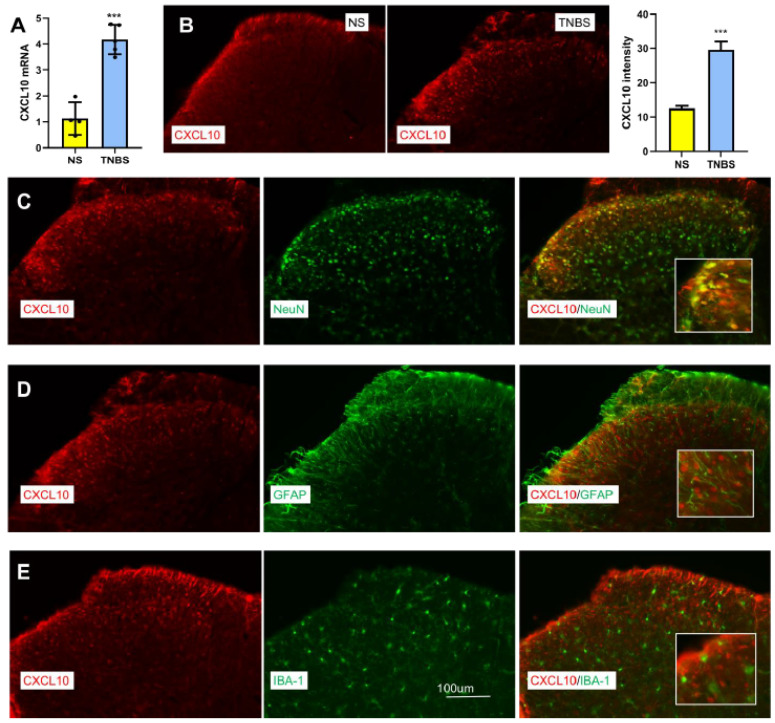
TNBS-induced upregulation of CXCL10 in spinal neurons. (**A**) TNBS significantly increased CXCL10 mRNA expression in the spinal cord (L6-S1), as determined by qPCR. ***, *p* < 0.001 vs. NS. Student’s *t*-test, *n* = 6. (**B**) Representative images and quantification showing increased CXCL10 immunoreactivity in the spinal dorsal horn of TNBS-treated mice. ***, *p* < 0.001 vs. NS. Student’s *t*-test. (**C**) CXCL10 (red) is predominantly colocalized with the neuronal marker NeuN (green) in the spinal dorsal horn. (**D**) CXCL10 (red) shows minimal colocalization with the astrocytic marker GFAP (green). (**E**) CXCL10 (red) shows minimal colocalization with the microglial marker IBA-1 (green).

In our previous study, we showed that mice exhibited significant visceral hyperalgesia 7 days after instillation of TNBS [24]. Here, we collected the spinal cord (L6-S1) at 7 days after TNBS to test the expression of CXCL10. qPCR and immunofluorescence revealed significant increases in spinal CXCL10 mRNA (Figure 1A) and protein levels (Figure 1B) following TNBS treatment, compared to the NS group. Co-localization analysis showed that CXCL10 was predominantly expressed in neurons (NeuN+, Figure 1C), with minimal overlap with astrocytes (GFAP, Figure 1D) or microglia (IBA-1, Figure 1E). These findings suggest that TNBS induces neuronal CXCL10 upregulation in the spinal cord, although predominantly observed in neurons under the present conditions.

### 3.2. TNBS Increases CXCR3 Expression Predominantly in Spinal Neurons

**Figure 2:**
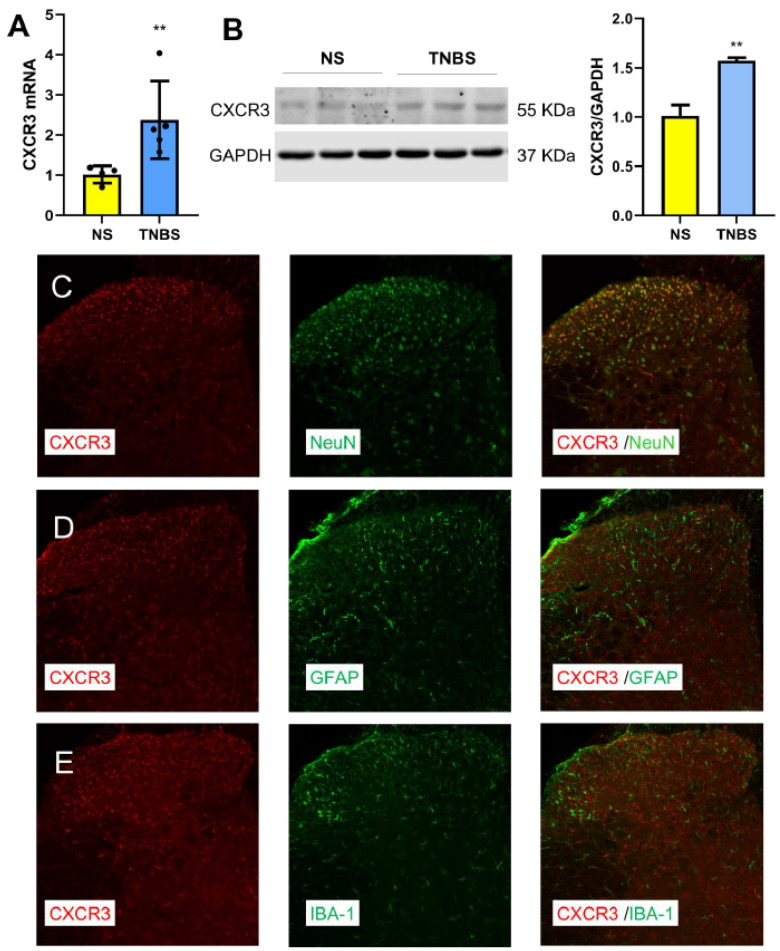
TNBS-induced upregulation of CXCR3 in spinal neurons. (**A**) TNBS significantly increased CXCR3 mRNA expression in the spinal cord (L6-S1), as determined by qPCR. **, *p* < 0.01 vs. NS. Student’s *t*-test, *n* = 6. (**B**) Western blot analysis and quantification showing increased CXCR3 protein expression in the spinal cord (L6-S1) of TNBS-treated mice compared with NS controls. **, *p* < 0.01 vs. NS. Student’s *t*-test. (**C**) CXCR3 (red) is predominantly colocalized with the neuronal marker NeuN (green). (**D**) CXCR3 (red) shows minimal colocalization with the astrocytic marker GFAP (green). (**E**) CXCR3 (red) shows minimal colocalization with the microglial marker IBA-1 (green).

In the same model, the CXCL10 receptor CXCR3 was also upregulated at the spinal cord level. qPCR and Western blot analyses showed that TNBS treatment significantly increased CXCR3 mRNA (Figure 2A) and protein levels (Figure 2B). Immunofluorescence revealed that CXCR3 was primarily localized to neurons in the dorsal horn (Figure 2C), with minimal co-localization with astrocytes (GFAP, Figure 2D) or microglia (IBA-1, Figure 2E). These findings suggest that TNBS induces neuron-specific CXCR3 upregulation in the spinal cord.

### 3.3. Spinal CXCL10 Is Associated with TNBS-Induced Visceral Hypersensitivity and ERK Phosphorylation

**Figure 3:**
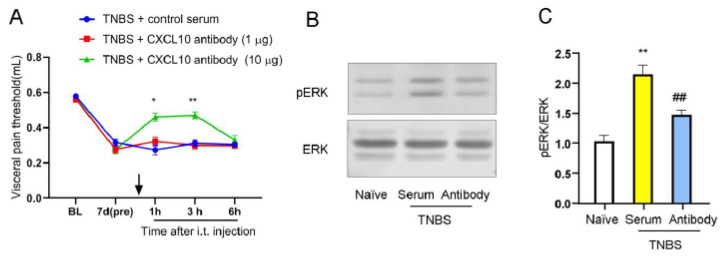
CXCL10 mediates visceral pain and spinal ERK activation in TNBS-treated mice. (**A**) Intrathecal administration of a CXCL10-neutralizing antibody (10 μg), but not 1 μg, significantly attenuated TNBS-induced visceral hypersensitivity at 1 h and 3 h after injection. The downward arrow indicates the administration time point. *, *p* < 0.05; **, *p* < 0.01 vs. TNBS + control serum, *n* = 6. (**B**,**C**) Western blot analysis and quantification showing that intrathecal CXCL10-neutralizing antibody (10 μg) markedly reduced TNBS-induced ERK phosphorylation in the spinal dorsal horn. **, *p* < 0.01 vs. naïve; ##, *p* < 0.01 vs. TNBS + serum.

To examine the role of CXCL10, neutralizing antibodies were administered intrathecally in TNBS-treated mice. The visceral pain thresholds were examined 1 h, 3 h, and 6 h after injection. Behavioral assays showed that 10 μg, but not 1 μg, of the antibody significantly attenuated TNBS-induced visceral hyperalgesia at 1 h and 3 h post-injection, compared with the control group (Figure 3A). At the molecular level, this intervention also markedly suppressed the TNBS-induced increase in spinal ERK phosphorylation (Figure 3B,C). These results are consistent with the involvement of spinal CXCL10 signaling in visceral hyperalgesia and its association with ERK pathway activation in this model.

### 3.4. Pharmacological Inhibition of CXCR3 Attenuates Visceral Pain and Spinal ERK Phosphorylation

**Figure 4:**
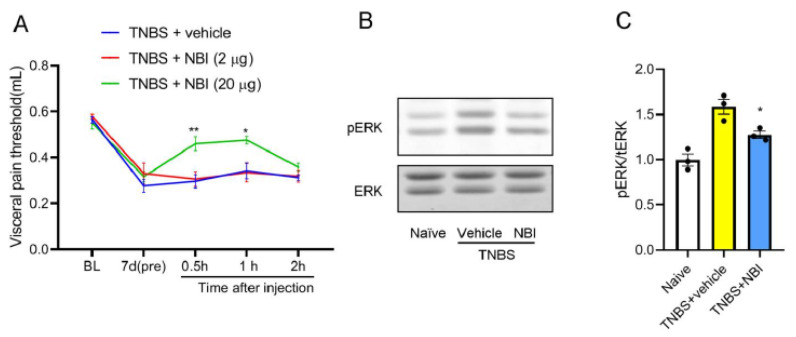
Intrathecal CXCR3 antagonist attenuates TNBS-induced visceral pain and spinal ERK activation. (**A**) Intrathecal administration of the CXCR3 antagonist NBI-74330 (20 μg), but not 2 μg, significantly attenuated TNBS-induced visceral hypersensitivity at 0.5 h and 1 h after injection. *, *p* < 0.05; **, *p* < 0.01 vs. TNBS + vehicle, *n* = 6. (**B**,**C**) Western blot analysis and quantification showing that intrathecal NBI-74330 (20 μg) markedly reduced TNBS-induced ERK phosphorylation in the spinal dorsal horn. *, *p* < 0.05 vs. TNBS + vehicle.

Given the coordinated upregulation of CXCL10 and CXCR3, the functional role of CXCR3 was further examined using pharmacological inhibition. NBI-74330, a selective and potent CXCR3 antagonist, was intrathecally injected 7 days after TNBS. We checked the visceral pain threshold 0.5 h,1 h, and 2 h after injection. As compared with the vehicle group, intrathecal administration of NBI-74330 (20 μg, but not 2 μg) significantly attenuated TNBS-induced visceral hypersensitivity at 0.5 h and 1 h (Figure 4A). Western blot of spinal tissue (L6-S1) concurrently showed that NBI-74330 (20 μg) suppressed spinal ERK phosphorylation (Figure 4B,C) in TNBS mice. These findings support the essential role of CXCR3 in mediating visceral pain and downstream ERK signaling activation.

### 3.5. CXCL10-Induced Visceral Hypersensitivity and ERK Activation Are Mediated by CXCR3

**Figure 5:**
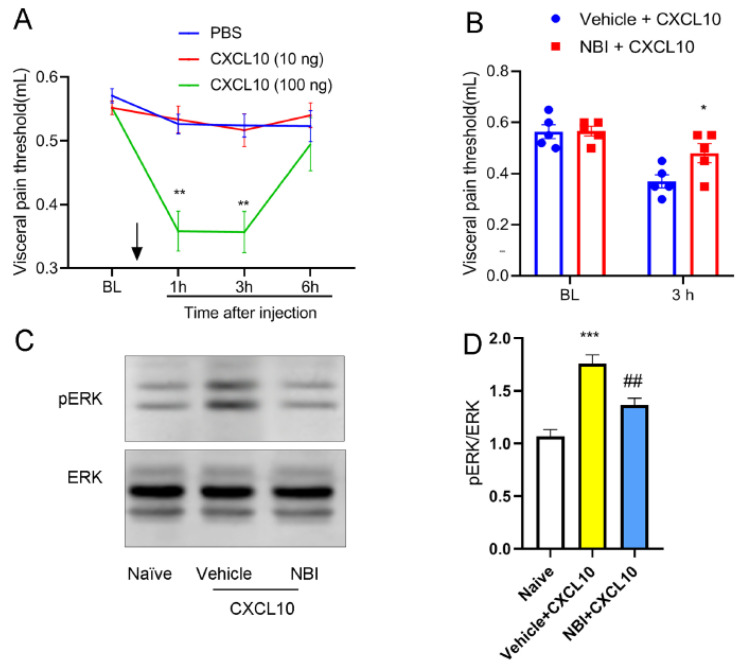
CXCL10-induced visceral hypersensitivity and spinal ERK activation are dependent on CXCR3. (**A**) Intrathecal administration of recombinant CXCL10 (100 ng), but not 10 ng, induced visceral hypersensitivity at 1 h and 3 h after injection in naïve mice. The downward arrow indicates the administration time point. **, *p* < 0.01 vs. PBS, *n* = 6. (**B**) Pretreatment with the CXCR3 antagonist NBI-74330 significantly attenuated CXCL10 (100 ng)-induced visceral hypersensitivity. *, *p* < 0.05 vs. vehicle + CXCL10, *n* = 6. (**C**,**D**) Western blot analysis and quantification showing that CXCL10-induced ERK phosphorylation in the spinal cord was markedly reduced by NBI-74330 pretreatment. ***, *p* < 0.001 vs. naïve; ##, *p* < 0.01 vs. vehicle + CXCL10.

To determine whether spinal CXCL10 directly induces pain, we examined pain behaviors in naive mice following intrathecal injection of recombinant CXCL10 protein. As compared with the vehicle (PBS) group, intrathecal administration of recombinant CXCL10 protein (100 ng, but not 10 ng) induced significant visceral pain hypersensitivity at 1 h and 3 h after injection (Figure 5A). To determine whether CXCL10 exerts its effects via CXCR3, naïve mice were first injected with CXCR3 antagonist NBI-74330, followed by administration of the recombinant protein 30 min later. Pain behaviors were then examined at 3 h. Pre-treatment with NBI-74330 effectively blocked both CXCL10-induced hypersensitivity (Figure 5B) and spinal ERK activation (Figure 5C,D). These results suggest that CXCL10 induces pain, and this effect is associated with CXCR3-dependent changes in spinal ERK phosphorylation.

### 3.6. Intrathecal DHA Alleviates TNBS-Induced Visceral Pain and Suppresses Spinal CXCL10/CXCR3/ERK Signaling

**Figure 6:**
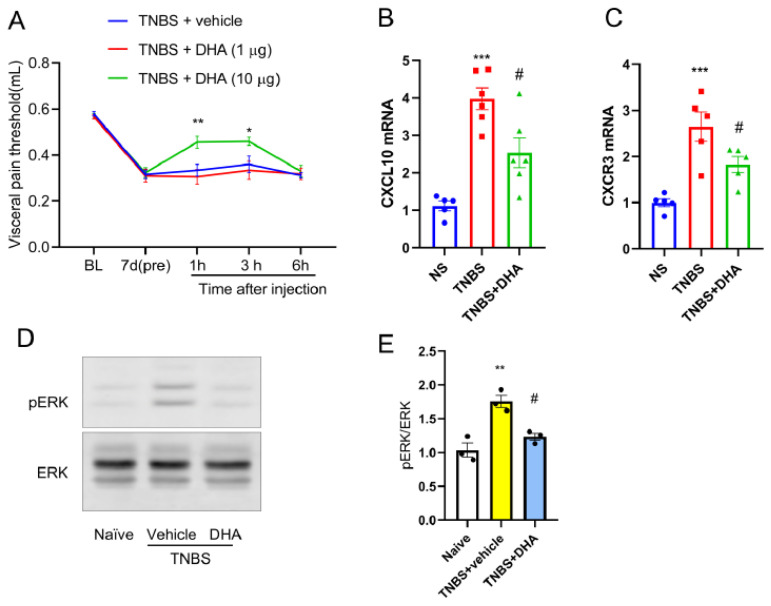
Intrathecal DHA attenuates TNBS-induced visceral pain and suppresses spinal CXCL10/CXCR3/ERK signaling. (**A**) Intrathecal administration of DHA (10 μg), but not 1 μg, significantly attenuated TNBS-induced visceral hypersensitivity at 1 h and 3 h after injection. *, *p* < 0.05; **, *p* < 0.01 vs. TNBS + vehicle, *n* = 6. (**B**,**C**) qPCR analysis showing that DHA treatment reduced TNBS-induced upregulation of CXCL10 and CXCR3 mRNA expression in the spinal cord. ***, *p* < 0.001 vs. naïve; #, *p* < 0.05 vs. TNBS, *n* = 6. (**D**,**E**) Western blot analysis and quantification demonstrated that DHA markedly suppressed TNBS-induced ERK phosphorylation in the spinal cord. **, *p* < 0.01 vs. naïve; #, *p* < 0.05 vs. TNBS + vehicle.

Based on the above findings, the intervention effect of DHA was further examined. On the seventh day after TNBS enema, different doses of DHA were administered intrathecally, and visceral pain behaviors were then examined at 1 h, 3 h, and 6 h. As compared with the vehicle group, intrathecal administration of DHA (10 μg, but not 1 μg) significantly attenuated TNBS-induced visceral hypersensitivity at 1 h and 3 h (Figure 6A). At the molecular level, DHA (10 μg) treatment suppressed the TNBS-induced upregulation of spinal CXCL10 and CXCR3 mRNA (Figure 6B,C) and reduced ERK phosphorylation (Figure 6D,E). These results suggest that the analgesic effect of DHA is associated with inhibition of the spinal CXCL10/CXCR3 axis and ERK signaling pathway.

### 3.7. DHA Inhibits Inflammatory CXCL10/CXCR3 Induction and ERK Activation in Neuronal Cells In Vitro

**Figure 7:**
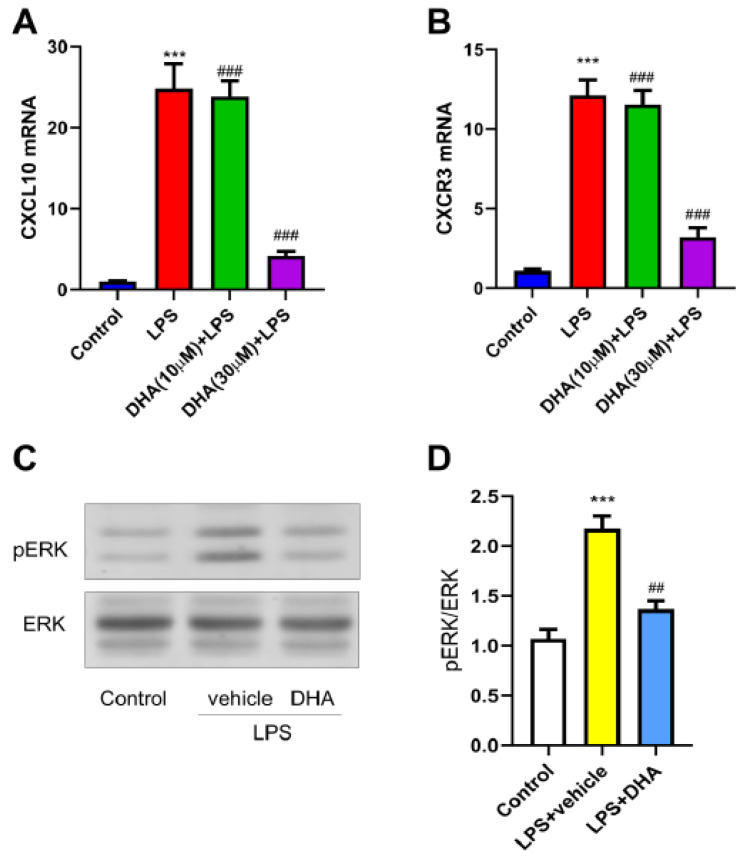
DHA attenuates LPS-induced CXCL10/CXCR3 expression and ERK activation in neuronal cells. (**A**,**B**) qPCR analysis showing that LPS markedly increased CXCL10 and CXCR3 mRNA expression in Neuro-2a cells, which was attenuated by DHA pretreatment in a dose-dependent manner. ***, *p* < 0.001 vs. control; ###, *p* < 0.001 vs. LPS alone. (**C**,**D**) Western blot analysis and quantification demonstrating that DHA pretreatment suppressed LPS-induced ERK phosphorylation in Neuro-2a cells. ***, *p* < 0.001 vs. control; ##, *p* < 0.01 vs. LPS + vehicle.

**Figure 8:**
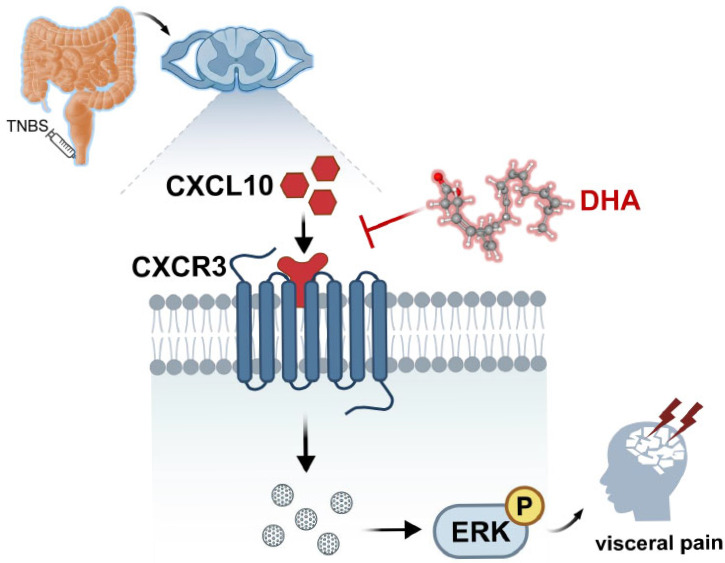
Mechanism of DHA-mediated visceral pain relief by spinal CXCL10/CXCR3/ERK pathway inhibition. The dot between CXCR3 and phosphorylated ERK represents intermediate signaling molecules in the signaling cascade, which mediate the signal transduction from CXCR3 activation to ERK phosphorylation.

To assess the direct cellular effects of DHA, in vitro experiments were performed using Neuro-2a cells. Cells were first pretreated with DHA(10 μmol/L, 30 μmol/L) for 30 min, followed by LPS stimulation for 6 h. Subsequently, the cells were collected for qPCR and Western blot analysis. LPS stimulation significantly increased CXCL10 and CXCR3 mRNA expression (Figure 7A,B) and enhanced ERK phosphorylation (Figure 7C,D). DHA pretreatment significantly attenuated these LPS-induced molecular changes in a dose-dependent manner, with the most notable effect at 30 μmol/L and only a modest effect at 10 μmol/L. The dot between CXCR3 and phosphorylated ERK represents intermediate signaling molecules in the signaling cascade, including G proteins, Ras, Raf, and MEK, which mediate the signal transduction from CXCR3 activation to ERK phosphorylation. These findings further support that DHA directly inhibits inflammation-induced activation of the CXCL10/CXCR3/ERK signaling axis at the neuronal level. Overall, the potential mechanism of DHA-mediated visceral pain relief involving spinal CXCL10/CXCR3/ERK pathway inhibition was summarized (Figure 8).

## 4. Discussion

Chemokines and their receptors are key contributors to central sensitization, mediating not only immune cell recruitment and activation but also acting as neuromodulators that directly regulate neuronal excitability and synaptic plasticity [8]. Recent evidence suggests that CXCL10, beyond its classical immunological role, modulates neuronal and glial function via CXCR3, influencing excitability, plasticity, and glial activation [9]. In the present study, CXCL10 and CXCR3 expression in the spinal dorsal horn were markedly upregulated in TNBS-induced visceral pain, closely paralleling the development of hyperalgesia. This temporal correlation suggests that CXCL10/CXCR3 signaling is involved in central sensitization associated with visceral pain. While previous work has primarily addressed its role in neuropathic pain [10], these findings extend the relevance of this pathway to visceral nociception, broadening the current understanding of chemokine-mediated pain mechanisms.

Cellular localization studies provide critical insights into the spatial and functional specificity of signaling pathways, particularly in the context of pain modulation. In the present study, using immunofluorescence double-labeling, we found that CXCL10 and its cognate receptor CXCR3 were predominantly expressed in neurons within the spinal dorsal horn under a visceral pain condition, while exhibiting minimal co-localization with markers of astrocytes and microglia. This distribution pattern, although predominantly observed in neurons under the present conditions, suggests that the CXCL10/CXCR3 signaling axis may involve neuronal populations in this context. Traditionally, spinal cord chemokines are thought to be chiefly produced by glial cells, mediating pain hypersensitivity through glia-to-neuron communication. For example, in neuropathic pain models, chemokines such as CCL2 and CXCL1 have been reported to activate CCR2 and CXCR2 receptors on neurons, thereby enhancing nociceptive transmission [8]. In contrast, our findings implicate neurons not only as recipients but also as potential sources of CXCL10. Neuronal CXCL10 may engage CXCR3 in an autocrine or paracrine manner, establishing a local positive feedback loop that amplifies and prolongs pain signaling. This novel neuron-centric mechanism suggests a departure from the canonical glial-dominant view of chemokine signaling in pain and introduces a previously underappreciated mode of intrinsic neuronal modulation. It is important to emphasize that these findings do not preclude the involvement of glial cells in visceral pain processing. Microglia and astrocytes may still contribute indirectly by releasing alternative mediators or influencing synaptic plasticity. Future investigations employing cell-type-specific manipulations will be critical to delineate the functional relevance of neuron-derived CXCL10 and its interplay with glial components during the initiation and maintenance of visceral pain.

Establishing a causal link between molecular signaling events and disease phenotypes lies at the heart of mechanistic pain research. In this study, we adopted a bidirectional validation strategy that integrates both loss- and gain-of-function approaches to elucidate the role of the CXCL10/CXCR3 axis in visceral pain. In the loss-of-function paradigm, intrathecal administration of a CXCL10-neutralizing antibody effectively sequestered endogenous CXCL10, preventing its engagement with CXCR3 and producing significant analgesic effects. This finding demonstrates the necessity of endogenous CXCL10 in sustaining visceral hypersensitivity. Consistently, administration of NBI-74330, a selective CXCR3 antagonist, also led to marked attenuation of visceral pain, corroborating the necessity of receptor-ligand interaction in mediating this process. The concordant outcomes from both interventions reinforce the conclusion that interruption of the CXCL10/CXCR3 interaction mitigates pain signaling. In the gain-of-function experiment, intrathecal injection of recombinant CXCL10 into naïve mice elicited robust visceral hypersensitivity, indicating that spinal activation of CXCL10 signaling may trigger the sensitization cascade. This pronociceptive effect was abrogated by co-administration of the CXCR3 antagonist, confirming that CXCL10 exerts its effect via CXCR3 rather than through alternative receptors or off-target mechanisms. Overall, these findings provide pharmacological evidence that CXCL10/CXCR3/ERK signaling contributes to visceral hypersensitivity, with pathway inhibition attenuating and exogenous ligand administration inducing pain behaviors.

Phosphorylation of ERK in spinal dorsal horn neurons is widely recognized as a key molecular marker of central sensitization and plays a pivotal role in the development and maintenance of various chronic pain [11]. In a previous study, it was confirmed that TNBS induced the activation of ERK in the spinal cord. The induction of visceral pain by TNBS was attenuated by the injection of ERK upstream kinase (MEK) inhibitor PD98059 [25]. In this study, we also checked that TNBS-induced visceral pain was associated with a significant increase in ERK phosphorylation in the spinal cord. Pharmacological blockade of the CXCL10/CXCR3 axis not only suppressed ERK activation but also produced significant analgesic effects, linking this chemokine pathway to ERK-mediated nociceptive processing. Further supporting this connection, intrathecal administration of recombinant CXCL10 in naïve animals triggered a marked increase in ERK phosphorylation, an effect that was abolished in the presence of a selective CXCR3 antagonist. These findings support a signal transduction cascade from CXCL10/CXCR3 engagement to downstream ERK activation. Mechanistically, previous studies have shown that CXCR3 activation can release the Gβγ subunit to initiate the canonical PI3K/Ras/Raf/MEK, ERK pathway [26]. CXCR3 may undergo GRK-mediated phosphorylation, subsequently recruiting β-arrestin to serve as a scaffold for non-canonical ERK activation [27]. It is plausible that both classical and β-arrestin-mediated pathways contribute to the CXCL10/CXCR3-driven ERK activation observed in this visceral pain model. Importantly, ERK phosphorylation is unlikely to be the sole downstream consequence of CXCR3 signaling. Parallel activation of other intracellular cascades, including p38 MAPK, JNK, and PI3K/Akt pathways, has been implicated in pain hypersensitivity and synaptic plasticity [28]. Future studies employing pathway-specific pharmacological inhibitors and genetic models will be essential to dissect the relative contributions of these parallel routes and to delineate the broader multimodal regulatory network governed by the CXCL10/CXCR3 axis in chronic pain.

The analgesic effects of omega-3 polyunsaturated fatty acids, particularly DHA, have been well reviewed [29]. DHA, a major component of membrane phospholipids in the nervous system, is particularly abundant in the brain and retina. Its analgesic action involves multiple mechanisms, including: modulation of cell membrane fluidity and lipid raft structure, competition with arachidonic acid metabolism to reduce pro-inflammatory mediator production, activation of anti-inflammatory signaling pathways, and promotion of the synthesis of specific inflammatory resolution mediators [30]. In this study, intrathecal DHA administration alleviated visceral hyperalgesia induced by TNBS, significantly reduced the upregulation of CXCL10 and CXCR3 mRNA in the spinal cord, and inhibited ERK phosphorylation. These results suggest that DHA exerts its analgesic effect potentially through modulation of the CXCL10/CXCR3/ERK signaling axis. The inhibitory effect of DHA on CXCL10 expression may involve transcriptional regulation. The CXCL10 gene promoter contains binding sites for multiple transcription factors, including NF-κB, STAT1, and IRF1. DHA has been shown to inhibit NF-κB activation through various mechanisms, including activation of PPARγ, which interferes with NF-κB DNA binding, and inhibition of TAK1 activation via the GPR120-β-arrestin2 complex [31]. Additionally, DHA has been reported to inhibit STAT1 phosphorylation and nuclear translocation [32], which may further contribute to the suppression of CXCL10 transcription. The regulation of CXCR3 expression by DHA is less well understood. The CXCR3 gene is regulated by transcription factors such as T-bet and STAT4 [33]. Whether DHA inhibits CXCR3 expression through modulation of these transcription factors, or by affecting mRNA stability or translation efficiency, warrants further investigation. DHA’s dual inhibition of both ligand and receptor offers a more comprehensive blockade of the CXCL10/CXCR3 signaling pathway, enhancing its potential as a therapeutic target for pain management.

In vivo experiments involve complex cellular networks and systemic regulation, making it difficult to determine whether DHA modulates neuronal CXCL10/CXCR3/ERK signaling directly or via indirect pathways. To address this, validation studies were conducted in the Neuro-2a neuronal cell line. DHA treatment inhibited LPS-induced upregulation of CXCL10 and CXCR3 expression and suppressed ERK phosphorylation, consistent with in vivo findings. Similar effects of DHA have also been reported in other models. Departing from the predominant focus on glial modulation, our study reveals a direct analgesic effect of DHA on neurons. These results confirm that DHA can act directly on neurons to modulate the CXCL10/CXCR3/ERK pathway, excluding confounding contributions from glial or immune cells in the in vivo context. Although the LPS stimulation model differs from the TNBS-induced model in upstream triggers, both converge on innate immune pathways such as TLR4/MyD88/NF-κB [34]. The consistent response to DHA across models suggests that its regulatory effects on the CXCL10/CXCR3/ERK axis may involve interference with shared upstream signaling nodes, strengthening the generalizability of these findings.

This study provides evidence with translational relevance for visceral pain management. As a member of the G protein-coupled receptor (GPCR) family, CXCR3 constitutes a pharmacologically tractable target with well-defined signaling properties. Our findings demonstrate that the selective CXCR3 antagonist NBI-74330 significantly attenuates visceral nociceptive responses, offering critical proof-of-concept for the development of CXCR3-targeted analgesics. Although CXCR3 antagonists have been primarily investigated in autoimmune diseases and transplant rejection [35], their application in pain modulation has remained largely unexplored. Equally important, this study highlights the analgesic potential of DHA, a safe and widely accessible omega-3 polyunsaturated fatty acid. While epidemiological data and clinical trials have reported inverse associations between omega-3 intake and various chronic pain syndromes, including rheumatoid arthritis, dysmenorrhea, and neuropathic pain [18], their impact on visceral pain remains insufficiently characterized. Here, we bridge this gap by establishing a mechanistic link between DHA administration and modulation of visceral pain signaling. Using complementary in vitro and in vivo approaches, we identify the spinal CXCL10/CXCR3/ERK signaling cascade as a key driver in the initiation and maintenance of visceral hypersensitivity. Importantly, our data show that DHA attenuates visceral pain through downregulation of this pathway, thereby providing mechanistic insight into its central analgesic action. Collectively, these findings deepen our understanding of central sensitization in visceral pain and pave the way for novel therapeutic strategies that combine pharmacological blockade of CXCR3 with dietary modulation.

This study has several limitations that warrant further investigation. First, the TNBS-induced colitis model primarily reflects visceral hypersensitivity associated with inflammatory bowel disease and may not fully recapitulate the immune and neurochemical profile of functional visceral pain conditions such as irritable bowel syndrome. Caution is therefore needed when extrapolating these findings across pain phenotypes. In addition, although this study, based on rodent models, provides crucial insights into the mechanisms of spinal central sensitization in visceral pain, the generalizability of its findings to human biology requires careful evaluation. There are inherent differences between animal models and humans in terms of visceral innervation, the specific composition of spinal signaling pathways, the intensity of neuro-immune interactions, and overall systemic complexity. These factors may influence the relative contribution and regulatory details of the CXCL10/CXCR3/ERK axis in human chronic visceral pain. Nevertheless, the key components of this signaling pathway are widely expressed in the human nervous system and immune cells and have been shown to be active in human neuropathic pain and inflammatory disorders, supporting the potential translational relevance of the present findings. Future studies should aim to validate the activity of this pathway in human visceral pain conditions using human spinal cord tissues, patient-derived cellular models, or noninvasive imaging techniques, thereby strengthening the linkage between these findings and human pain management. Second, the current work focuses on acute and subacute phases of visceral pain. The temporal dynamics of CXCL10/CXCR3/ERK signaling in chronic states, as well as the long-term analgesic efficacy of DHA, remain to be elucidated. Third, while intrathecal delivery clearly demonstrates a spinal mechanism of DHA action, its translational relevance is limited. Future studies should evaluate the pharmacokinetics, permeability across the blood-spinal barrier, and therapeutic efficacy of DHA under systemic administration (oral or intravenous) to facilitate clinical translation. Finally, one limitation of this study is the lack of detection of specific DHA metabolites. Consequently, it remains unclear whether intrathecally administered DHA acts directly or indirectly through metabolites such as resolvins. Addressing these gaps will advance understanding of the neuroimmune actions of DHA and support its development as a clinically applicable analgesic strategy.

## 5. Conclusions

This work suggests that the spinal CXCL10/CXCR3/ERK signaling axis plays a critical role in the central sensitization associated with visceral pain. By employing systematic in vitro and in vivo approaches, we demonstrate that spinal neuron CXCL10/CXCR3 signaling is necessary and plays a central role in driving visceral nociception. ERK phosphorylation appears to act as a key mediator, linking receptor activation to neuronal plasticity. Importantly, a novel mechanism was elucidated whereby DHA may exert its analgesic effects involving multi-target inhibition of this pro-nociceptive signaling pathway. These findings provide not only a deeper mechanistic understanding of visceral pain but also identify potential therapeutic targets and support the molecular rationale for the clinical application of DHA in pain management.

## Data Availability

The data that support the findings of this study are available in the manuscript. Extra data used to support this study are available from the corresponding author upon request.
